# The contradictory roles of macrophages in non-alcoholic fatty liver disease and primary liver cancer—Challenges and opportunities

**DOI:** 10.3389/fmolb.2023.1129831

**Published:** 2023-02-10

**Authors:** Marlene Sophia Kohlhepp, Hanyang Liu, Frank Tacke, Adrien Guillot

**Affiliations:** Department of Hepatology and Gastroenterology, Charité Universitätsmedizin Berlin, Campus Virchow-Klinikum and Campus Charité Mitte, Berlin, Germany

**Keywords:** liver macrophages, hepatocellular carcinoma, immunotherapy, non-alcoholic steatohepatitis, monocytes, tumor-associated macrophages

## Abstract

Chronic liver diseases from varying etiologies generally lead to liver fibrosis and cirrhosis. Among them, non-alcoholic fatty liver disease (NAFLD) affects roughly one-quarter of the world population, thus representing a major and increasing public health burden. Chronic hepatocyte injury, inflammation (non-alcoholic steatohepatitis, NASH) and liver fibrosis are recognized soils for primary liver cancer, particularly hepatocellular carcinoma (HCC), being the third most common cause for cancer-related deaths worldwide. Despite recent advances in liver disease understanding, therapeutic options on pre-malignant and malignant stages remain limited. Thus, there is an urgent need to identify targetable liver disease-driving mechanisms for the development of novel therapeutics. Monocytes and macrophages comprise a central, yet versatile component of the inflammatory response, fueling chronic liver disease initiation and progression. Recent proteomic and transcriptomic studies performed at singular cell levels revealed a previously overlooked diversity of macrophage subpopulations and functions. Indeed, liver macrophages that encompass liver resident macrophages (also named Kupffer cells) and monocyte-derived macrophages, can acquire a variety of phenotypes depending on microenvironmental cues, and thus exert manifold and sometimes contradictory functions. Those functions range from modulating and exacerbating tissue inflammation to promoting and exaggerating tissue repair mechanisms (i.e., parenchymal regeneration, cancer cell proliferation, angiogenesis, fibrosis). Due to these central functions, liver macrophages represent an attractive target for the treatment of liver diseases. In this review, we discuss the multifaceted and contrary roles of macrophages in chronic liver diseases, with a particular focus on NAFLD/NASH and HCC. Moreover, we discuss potential therapeutic approaches targeting liver macrophages.

## 1 Introduction

The liver is a vital organ with essential metabolic and immunological functions. Located at the crossroads between the systemic and the gut-derived blood circulation, it represents a privileged site for multifactorial inter-organ interactions. Furthermore, and due to its particular histological features, the liver is an organ with intense cell-cell interactions. A typical multifactorial condition is non-alcoholic fatty liver disease (NAFLD). (Peiseler et al., 2022) NAFLD is defined by hepatic steatosis (accumulation of fat in hepatocytes) independent from alcohol consumption or other medical conditions, it may progress to non-alcoholic steatohepatitis (NASH, incidence 2%–5% in the general population) and liver cirrhosis. Due to the global increase of obesity and metabolism-related diseases, NAFLD/NASH is expected to become the prime risk factor for hepatocellular carcinoma (HCC). (Ioannou, 2021; Riazi et al., 2022) Primary liver cancers are the third leading cause of cancer-related death worldwide despite only ranking as the sixth most frequently diagnosed cancer overall. (Llovet et al., 2021; Sung et al., 2021) Reasons for the high mortality are that liver cancer is often diagnosed at an advanced stage when resection or transplantation, the only curative approaches, are not options anymore. HCC is the most prevalent form of primary liver cancer and predominantly develops in fibrotic or cirrhotic livers in a setting of chronic inflammation. (Forner et al., 2012) A recent study predicted that the incidence of liver cancer will increase by 55.0% between 2020 and 2040, with a predicted 1.3 million deaths from liver cancer in 2040 (+56.4% compared to 2020) globally. (Rumgay et al., 2022a) The main reason for the expected increased incidence of HCC is the NAFLD/NASH epidemic, urging for a better understanding of the underlying mechanisms of hepatocarcinogenesis in this metabolic and inflammatory condition. (Riazi et al., 2022) Noteworthy, and mainly due to unhealthy life habits, about one-quarter of the world population presents varying degrees of NAFLD. Despite recent advances in liver disease understanding, therapeutic options for NAFLD patients remain limited and there is currently no effective therapeutic option for HCC. (Llovet et al., 2021) Thus, there is an urgent need to identify targetable liver disease-driving mechanisms for the development of novel therapeutics.

The liver may to some extent be regarded as an immunological organ, as it serves as the primary gateway for gut-derived (food- or microbiota-derived) antigens and is densely populated with immune cells, most notably myeloid cells in forms of liver resident macrophages during homeostasis, also named Kupffer cells (KCs). It is important to point out that KCs roughly represent 15% of total liver cells, pointing towards their central contributions to organ functions. (Lopez et al., 2011) Nonetheless, the remarkable diversity of liver myeloid cells was previously overlooked, both during homeostasis and (pre-) malignant liver diseases. Indeed, recent studies using multiplexed proteomic or transcriptomic studies highlighted the heterogeneity of the macrophage compartment, as exemplarily pointed out by Mulder et al. as part of the “monocyte and macrophage universe” (MoMacs-verse). (Mulder et al., 2021) Upon acute and chronic liver diseases, and this is particularly true in NAFLD/NASH, the “liver macrophage” compartment undergoes drastic changes both in terms of cellular origin, and in terms of phenotypic activation. Those changes primarily correspond to protective mechanisms against, for instance, pathogens or metabolism-related tissue injury, but are also responsible for disease progression when exacerbated. In this review, we aim at providing a current state-of-the-art view on the monocyte/macrophage landscape in healthy and diseased liver, and how this influences liver malignancies.

## 2 The manifold faces of “liver macrophages” in the liver at steady state

When discussing the roles of liver macrophages, it is important to firstly define what we refer to as “liver macrophages”. Indeed, in recent years a certain number of dogmas were challenged by multiplexed and high-dimensional approaches such as single liver cell transcriptome analysis, flow cytometry, cell tracing and multiplex immunohistochemistry. These approaches revealed a previously underestimated diversity and heterogeneity of the liver macrophage pool at a given time in disease course, as well as at a given location in the liver.

### 2.1 The liver sentinels: Kupffer cells

Viewing the liver as an immunological organ acknowledges its central role in controlling local and systemic immune responses, notably through the release into the main bloodstream of alarmins and complement factors by hepatocytes. (Bogdanos et al., 2013) Most importantly, a yolk sac derived macrophage population remains in the liver and gives rise to self-renewing liver resident macrophages, the KCs. In the adult liver, KCs are located within the sinusoid capillaries and present large cytoplasmic expansions, and are thus ideally located to sense changes in the circulating blood. KCs exert key functions in liver homeostasis maintenance, and tolerance to harmless food- or gut-derived antigens entering the liver *via* the portal vein, as well as clearing the systemic blood from exogenous or endogenous particles (e.g., pathogens, dead cell debris, red blood cells). (David et al., 2016; Gola et al., 2021; Guilliams et al., 2022) KCs were also shown to directly control T cell activation, although not as efficiently as dendritic cells and most often as a tolerogenic mechanism. (You et al., 2008; Heymann et al., 2015) KCs also possess the ability to repress dendritic cell-induced T cell activation, as demonstrated by lower T cell proliferation when KCs were introduced into T cell and dendritic cell co-cultures. In line, earlier studies demonstrated that the liver is a privileged site for CD8^+^ T cell apoptosis. (Huang et al., 1994; Bertolino et al., 1995) On the other hand, KCs also act as the immune system sentinels, and are among the first responders to pathogen- or damage-associated molecular patterns (PAMPs and DAMPs, respectively). Upon liver injury and despite their immunotolerant functional roles, KCs represent a major source of chemoattractants for circulating immune cells, including bone-marrow derived macrophages. Similarly to other immune cell populations, multiple KC subtypes have been identified, with varying functions during homeostasis and disease. (Bleriot et al., 2021; Guilliams et al., 2022)

### 2.2 The task force: Monocyte-derived macrophages

Monocyte-derived macrophages (MoMFs) represent a complex yet very intriguing compartment of the innate immune system, as naïve and freshly recruited MoMFs can rapidly accumulate at the injury sites and be directed towards a plethora of activated phenotypes depending on microenvironment-derived signals, ranging from pro- to anti-inflammatory, and pro- to anti-fibrotic. MoMFs can also either exacerbate pathological processes leading to tissue injury or play crucial roles in supporting tissue repair. Recent findings from single cell transcriptome sequencing and spatial proteomics evidenced the diversity of MoMF phenotypes even at a given time in a singular tissue, and will be detailed below with a particular focus on liver cancer. (Ramachandran et al., 2019; Krenkel et al., 2020; Guilliams et al., 2022; Hundertmark et al., 2022) Thus, MoMF populations represent very dynamic and astonishingly flexible immune cells. A specific MoMF population was shown to be present preferably around bile ducts during homeostasis, identified as *Gpnmb*-expressing and termed lipid-associated macrophages (LAMs), in reference to the monocytes that were shown to accumulate during liver steatosis. (Remmerie et al., 2020; Guilliams et al., 2022)

### 2.3 Additional sources of liver macrophages

Peritoneal macrophages represent an alternative source of macrophages, as described by Wang and Kubes in a mouse model of focal and superficial thermal-induced liver injury. (Wang and Kubes, 2016) In this study, the authors described a non-vascular route of macrophage infiltration towards the injury sites, and an active participation of GATA6-positive peritoneal macrophages in inducing tissue repair. The authors hypothesize peritoneal macrophages migrating through the liver capsule may be implicated in liver cancer as well, but this remains to be demonstrated. Similarly, some studies demonstrated the presence of capsular area-specific macrophages. The capsular macrophages were defined as negative for CLEC4F and TIMD4 but positive for CD11b and F4/80 in mice. (David et al., 2016; Sierro et al., 2017) Peritoneal macrophages were also shown to express CD207, similarly to some macrophages located at the central vein. (Guilliams et al., 2022) A function attributed to the capsular macrophages was to limit the propagation of peritoneal pathogens to the liver, by inducing neutrophil recruitment to the liver. (Sierro et al., 2017) Overall, the implications of capsular macrophages in NAFLD and HCC remain to be elucidated.

## 3 The sword of damocles: Focus on the roles of liver macrophages in NAFLD/NASH as a pre-malignant condition

Primary liver cancers are mainly of two cellular origins: hepatocytes (leading to HCC, which accounts for 80% of primary liver cancers), or cholangiocytes (leading to cholangiocarcinoma, CCA, 15% of primary liver cancers). (Rumgay et al., 2022b) Liver cirrhosis or even chronic liver diseases at earlier stages, are regarded as major risk factors for HCC. On the other hand, risk factors for CCA remain to be clarified, since CCA has higher risks to arise in healthy livers without any apparent risk factor as compared to HCC. (Banales et al., 2020) NAFLD encompasses a large range of chronic liver disease conditions, notably characterized by varying degrees of steatosis (i.e., excess fat accumulation in hepatocytes), inflammation, and fibrosis. In the last decades, much knowledge has been gathered that demonstrate liver macrophage implications in virtually all the aspects of NAFLD initiation and progression, comforting earlier hypotheses of multiple parallel hits. Nevertheless, the multifaceted yet contradictory macrophage functions render macrophage-targeting strategies challenging. For all these reasons, this section describes the current knowledge on the intricate roles of liver macrophages that may have pro- or anti-tumoral roles in NAFLD- and NASH-associated HCC. (Ueno et al., 2022)

### 3.1 An adapting (im) balance between resident KCs and MoMFs mobilization and spatial distribution

The healthy liver is primarily populated by liver resident macrophages (KCs) and patrolled by MoMFs. We and others have demonstrated that in mouse models of NAFLD/NASH, there is a global disruption in the liver macrophage compartment, notably visualized by a massive infiltration of MoMFs and a reduction in KC numbers. (Heymann et al., 2015; Reid et al., 2016; Guillot et al., 2020; Mulder et al., 2021) The changes observed in the balance between the distinct macrophage populations have dramatic implications for the local immune milieu, considering the major functional differences attributed to specific macrophage pools (i.e., KCs *versus* MoMFs). The histological changes observed during NAFLD progression also include a marked accumulation of immune cells, notably MoMFs, within the peri-lobular areas, marked with active fibrogenesis and inflammation. (Guillot et al., 2023) This has several consequences on the inflammatory status of the liver, especially considering that the portal areas represent the primary sites of entry for gut-derived antigens, usually captured by KCs without the initiation of an inflammatory response, as opposed to MoMFs that are prone to respond to bacterial antigens by the secretion of pro-inflammatory mediators. (Knolle et al., 1995) Thus, the disturbed KC/MoMF balance together with a preferential localization of MoMFs in portal areas, may further enhance a sustained and detrimental inflammation in the liver.

### 3.2 Liver macrophages as inflammation orchestrators

KCs are regarded as the liver sentinels, generally playing the role of immunotolerant cells that prevent excessive inflammation to harmless antigens. For instance, it is long-known that KCs respond to lipopolysaccharides by releasing IL-10, thus reducing the local secretion of pro-inflammatory IL-6 and tumor necrosis factor-alpha (TNF-α). (Knolle et al., 1995) However, the transcriptomic profiles of myeloid cells present in the liver, but also their precursors found in the bone marrow, were shown to be drastically altered and for a prolonged time upon steatohepatitis. This was notably demonstrated by single cell sequencing that showed a reduced *S100a9* ^+^ myeloid cell population in Western diet fed mice. In the same study, bone marrow transfer from normal chow to Western diet fed mice resulted in increased liver injury upon exposure to acetaminophen, revealing a potentially protective macrophage phenotype against excessive inflammatory response to hepatic injury. (Krenkel et al., 2020) Accordingly, it was shown that macrophage depletion prior to or when starting a methionine/choline deficient diet model of NASH, meaning at a point where the liver is mostly populated with KCs, resulted in decreased steatosis and monocyte recruitment to the liver. (Tosello-Trampont et al., 2012; Reid et al., 2016) Guilliams et al. suggested a rather anti-inflammatory role of LAMs, which also accumulate in a mouse NAFLD model. (Guilliams et al., 2022) Indeed, in this study, the authors showed that LAMs found in Western diet fed mice had lower gene expression of pro- (e.g., *Tnfa*, *Il1b*) and anti-inflammatory (e.g., *Il10*) cytokines compared to their counterparts found in the healthy liver. Furthermore, the authors reported on the presence of similar macrophage phenotypes in human steatotic liver, although this was observed in a limited number of patient samples and needs further validation, particularly on establishing the functional relevance of this macrophage heterogeneity. Besides, liver macrophages were shown to respond to, and also to direct, T cell response in homeostasis, but also during liver disease. (Mulder et al., 2021) More specifically, macrophages play central roles in amplifying interleukin-17A (IL-17A)-driven inflammation and fibrosis in the liver. (Guillot et al., 2014) Indeed, IL-17A-receptor is ubiquitously expressed and has been shown to have multiple roles in inflammatory disease progression. IL-17A treatment directly increased pro-inflammatory cytokine expression on macrophages, and enhanced myofibroblast collagen expression.

### 3.3 Liver macrophage roles in the installment of liver steatosis

KCs and macrophages in general, are characterized by their ability to sense and phagocytose particles or molecules in their surrounding environment. As such, toll-like receptors (TLRs) represent a family of membrane or cytoplasmic receptors triggering signaling cascades responsible for directing further immune responses. TLR4 is a known receptor for lipopolysaccharides, and its activation leads to the release of potent pro-inflammatory cytokines. Furthermore, TLR4 also binds free fatty acids, leading to NF-κB activation and TNF-α/IL-6 release. (Shi et al., 2006) Noteworthy, macrophages may actively influence the global metabolism. This was for instance evidenced by a manuscript from Jourdan et al., in which KC-specific cannabinoid-receptor type 1 deficient obese mice had improved glucose tolerance and insulin sensitivity yet similar liver fat content compared to their wild-type counterparts. (Jourdan et al., 2017) These effects were attributed to an *Il-6*, *Ccl2* and *Tnf-α* gene expression reduction by KCs in obese mice, and reduced oxidative stress in KCs. In the same study, conditioned medium from KCs inhibited the hepatocyte response to insulin, which was abrogated in cannabinoid receptor type 1 deficient KCs. Lipid-associated macrophages (LAMs) have been described as a population of macrophages accumulating in the adipose tissue of obese humans and mice and displaying lipid metabolism and phagocytosis related gene signatures. Lipid accumulation in these metabolically active macrophages was further evidenced by staining of neutral lipids with BODIPY, CD9 and TREM2 staining. (Jaitin et al., 2019) Similarly, MoMFs isolated from Western diet fed mice were shown to have decreased *S100a8* and *S100a9* expression but increased *Plin2*, suggesting MoMFs are similarly implicated in lipid metabolism. (Krenkel et al., 2020) In the liver, LAMs have been described as *Trem2*-expressing macrophages observed in a high-fat diet murine model and in NASH and obese patients. (Jaitin et al., 2019; Hendrikx et al., 2022) Furthermore, a large body of evidence demonstrated that TREM2^+^ macrophages have an anti-inflammatory role. (Jaitin et al., 2019; Remmerie et al., 2020; Zhang et al., 2022; Zhou et al., 2022) Conversely, Ramachandran et al. described scar-associated macrophages in human cirrhotic liver that co-expressed LAM signature genes, such as *TREM2*, *GPNMB*, *CD9* and *SSP1*, but also mitogens for fibroblasts such as *PDGFB* and *TNSFS12* (TWEAK) and displayed a pro-fibrotic phenotype. (Ramachandran et al., 2019) TREM2 is a transmembrane receptor of the immunoglobulin superfamily that recognizes lipids and apolipoproteins, and promotes immune tolerance during NAFLD. (Colonna, 2003) Mechanistically, TREM2 is associated with and signals *via* DAP12, which downregulates the transcription of inflammatory genes like *TNFA, IL1B*, and *NOS2*. (Takahashi et al., 2007) TREM2^+^ macrophages seem to be a highly conserved population, as macrophages with closely similar gene signatures have been described in different tissues and diseases, such as adipose tissue, atherosclerosis and Alzheimer disease. (Jaitin et al., 2019) Thus, TREM2 has emerged as a marker for an immunosuppressive lipid-associated macrophage subset in fatty liver. (Jaitin et al., 2019; Remmerie et al., 2020; Zhang et al., 2022) The fatty acid translocase CD36 is a scavenger receptor that binds and internalizes fatty acids and lipoproteins and can stimulate pro-inflammatory as well as anti-inflammatory functions in macrophages. In atherosclerosis and fatty liver disease, internalization of modified lipids, such as oxidized LDL, by CD36-expressing macrophages resulted in formation of inflammatory foam cells. (Rahaman et al., 2006; Bieghs et al., 2012) Interestingly, the pro-inflammatory functions of CD36 are dependent on co-activation of TLRs. On the contrary, CD36 expression is increased on restorative macrophages and participates in anti-inflammatory functions, like efferocytosis and the uptake of fatty acids, acting as ligands for anti-inflammatory nuclear receptors (i.e., PPARs) or substrate for fatty acid oxidation. (Canton et al., 2013; Puengel et al., 2022a) Furthermore, *Cd36* targeted silencing in KCs but not in MoMFs led to decreased liver oxidative stress (reduced malondialdehyde and reactive oxygen species accumulation) although this did not affect liver total triglyceride levels in obese mice. (Bleriot et al., 2021)

### 3.4 The impact of liver macrophages on fibrogenesis

The progression of liver diseases is typically characterized by the extent of liver fibrosis. Liver fibrosis is defined by the excessive accumulation of extracellular matrix (ECM) proteins, predominantly produced by activated hepatic stellate cells (HSC) or myofibroblasts. Other mesenchymal cell populations such as portal fibroblasts may contribute to ECM production as well. (Lei et al., 2022) Liver macrophages, including both KCs and MoMFs, represent major sources of HSC-/myofibroblast-activating cytokines including TGF-β, TNF-α, IL-1β and IL-6. (Pradere et al., 2013; Trautwein et al., 2015) Oncostatin M (OSM) is another pro-fibrotic cytokine released by macrophages, that promotes liver fibrosis particularly by inducing the expression of tissue inhibitor of metalloproteinase 1 (Timp-1). (Matsuda et al., 2018) This study notably showed that in the absence of underlying tissue injury, OSM overexpression is sufficient to initiate liver fibrogenesis. This fibrogenesis was attributed to the strong accumulation of MoMFs in the fibrotic areas and higher IL-6, TNF-α, and IL-1ß levels in the liver. Macrophage Mer Tyrosine Kinase (MerTK) has also been shown to play an indirect role in HSC activation in NASH, notably by inducing ERK1/2 phosphorylation and TGF-β1 release by KCs after Gas6 stimulation. (Cai et al., 2020) Moreover, this study also described that macrophage MerTK induced palmitate-treated hepatocyte cell death through TGF-β, a mechanism potentially aggravating not only liver fibrosis but also tissue injury in NASH. In accordance with the contradictory functions of liver macrophages, it was shown that increased TREM2^+^ macrophage recruitment in fibrotic NASH livers and higher soluble TREM2 levels in circulating blood, were associated with better outcome in patients, suggesting protective functions of TREM2^+^ macrophages notably through lipid-metabolism regulatory functions. (Ramachandran et al., 2019; Hendrikx et al., 2022) Interestingly, TREM2^+^ macrophages accumulate in the tissue areas with active fibrogenesis, oxidative stress and inflammation. Recent developments have allowed us to go beyond the “classical”, well-established molecular crosstalk between liver macrophages and fibrogenic cells, and new therapeutic targets of interest for fibrosis resolution through macrophage phenotype modulation towards a fibrolytic phenotype, or targeting pathways that are specific for fibroblast activation and proliferation (for example PDGFRA and TNFSF12A on scar-associated mesenchymal cells) are expected to be identified in the near future. (Krenkel et al., 2019; Ramachandran et al., 2019; Ramachandran et al., 2020; Cheng et al., 2021; Guilliams et al., 2022; Lee et al., 2022; Tada et al., 2022) Importantly, most macrophage-derived cytokines or macrophage polarizing factors affect multiple cell populations. This is particularly exemplified by IL-17A, which was shown to exert potent pro-inflammatory and fibrogenic effects, by acting directly on virtually all liver and immune cells. (Guillot et al., 2014; Li et al., 2021) Intriguingly, it has been suggested that the combined measurement of high alpha-foetoprotein and IL-17A could be predictive of future HCC development in cirrhotic livers. (Liang et al., 2021)

## 4 Turning the steatohepatitis-driven immune activation into a tumor promoting environment

**FIGURE 1:**
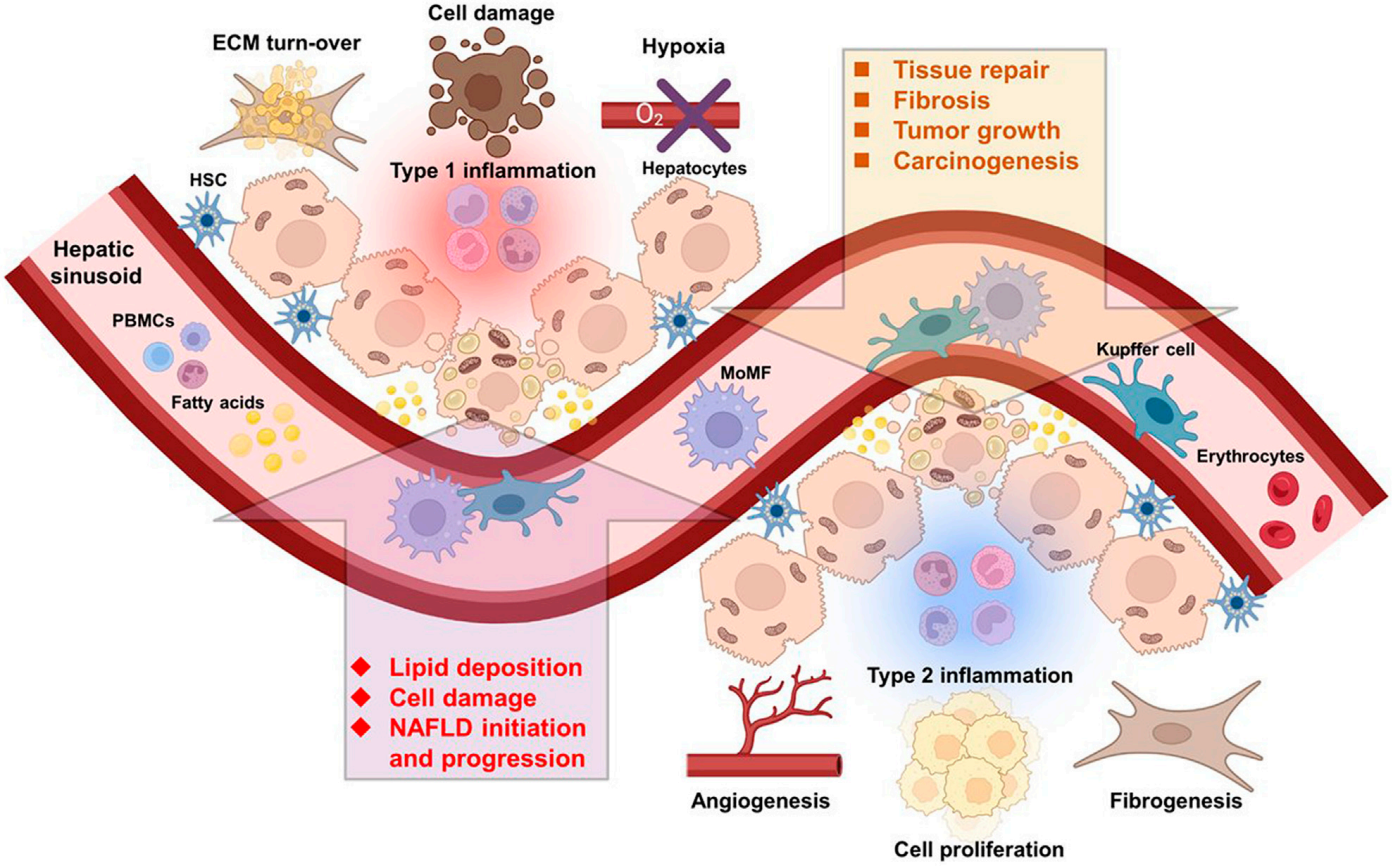
Immune cells of multiple origins influence the course of NAFLD and HCC. Liver resident and infiltrating immune cells from the circulation are directly implicated in NAFLD and HCC initiation and progression. Abbreviations, ECM, extracellular matrix; HSC, hepatic stellate cell; MoMF, monocyte-derived macrophage; NAFLD, non-alcoholic fatty liver disease; PBMCs, peripheral blood mononuclear cells. Created with BioRender.com.

Macrophages display an astonishingly high plasticity and ability to adapt to environmental cues in order to react to a variety of unfavorable conditions threatening the organism. The tumor stroma also called the tumor microenvironment (TME) exhibits peculiar conditions that conveniently coerce the macrophage phenotype towards a tumor-promoting state (Figure 1). In this section, we will discuss the TME specificities, and how it is shaped by and influences macrophages.

### 4.1 The tumor microenvironment influences macrophage functions

#### 4.1.1 The fibrotic and steatotic neoplastic niche—shaping the macrophage milieu?

Advanced liver fibrosis, a pathological wound-healing reaction towards chronic liver injury and inflammation, represents a considerable risk factor for HCC. (Li et al., 2021) Indeed, about 90% of all HCC develop in the background of a fibrotic or cirrhotic liver, characterized by excess ECM. (58) The functions of the ECM go beyond merely providing structural support. The composition of the ECM is dynamic and complex and can influence adhesion, signaling and proliferation of the adjacent cells. Importantly, increased matrix stiffness induces signaling pathways driving HCC development. (Levental et al., 2009) The hepatic TME is enriched in activated fibroblasts mainly originating from HSCs. (Affo et al., 2017) In a recent study, Filliol et al. described two subsets of HSCs with dual roles in mouse models of HCC. On the one hand, collagen-I producing activated myofibroblastic HSCs that promoted proliferation and tumor development through activation of TAZ in premalignant hepatocytes and discoidin domain receptor 1 in tumors. A subset of cytokine-producing and quiescent HSCs, on the other hand, carried out a tumor-limiting role by producing protective mediators such as hepatocyte growth factor (HGF). Interestingly, collagen-I producing fibroblasts accumulated predominantly around the HCC nodules but not within the tumor, suggesting their predominant role in establishing a tumor promoting preneoplastic niche. (Filliol et al., 2022) Activated HSCs are not only the main producers of ECM in the liver but also a source of TGF-β, and chemokines like CCL2 that attract CCR2^+^ monocytes. (Levental et al., 2009; Affo et al., 2017) Besides recruitment, activated HSCs are also implicated in skewing macrophage phenotype towards immunosuppression. (Ji et al., 2015) This is at least in part mediated through direct cell-cell contact between HSCs and monocytes involving CD44, as shown *in vitro* using CD14^+^ human blood monocytes. (Hochst et al., 2013). In this study, the authors demonstrated that coculture with, but not conditioned medium from activated HSCs rendered human blood monocytes immunosuppressive, characterized by reduced HLA-DR expression and the ability to suppress CD8 T cell proliferation through arginase 1 (ARG1), an effect that could be abrogated by CD44 blockade. CD44 proteins belong to a family of ubiquitously expressed cell surface adhesion proteins, and are important mediators of cell-cell contact and adhesion but also regulate many biological activities. CD44 binds to several extracellular matrix proteins, among others hyaluronic acid, collagens and osteopontin. (Senbanjo and Chellaiah, 2017) Hepatic macrophages, on the other hand, can activate quiescent HSCs *via* TGF-β and promote survival of myofibroblasts through the secretion of IL-1 and TNF, thereby facilitating fibrosis (Pradere et al., 2013; Fabregat and Caballero-Diaz, 2018) In a study on patients with non-viral HCC, the TME of steatotic HCC subtypes was enriched in immune cells and cancer-associated fibroblasts (CAFs), alongside with an increased *CCL2* expression and an over activated TGF-β pathway compared to other HCC subclasses. (Murai et al., 2022) Similarly, TGF-β was upregulated in diet-induced NASH-HCC in mice. Interestingly, TREM2^+^ LAMs expressed *Tgfrb1* and *in vitro* stimulation of murine bone marrow derived macrophages with TGF-β induced *Trem2* mRNA expression, along with other TREM2^+^ macrophage markers such as *Gpnmb* and *Tgfbr1*. (Zhang et al., 2022) Activated fibroblasts from steatotic and fibrotic livers and CAFs are not only a source of CCL2 and involved in the recruitment of CCR2^+^ myeloid cells (such as TREM2^+^ macrophages), but also produce TGF-β, suggesting a link between the accumulation TREM2^+^macrophages in fibrosis, steatosis and HCC. (Yang et al., 2016; Affo et al., 2017; Schwabe et al., 2020; Guilliams et al., 2022)

Even though most NASH-HCCs arise in a cirrhotic liver, HCC can also develop in steatotic livers without advanced fibrosis or cirrhosis. (Paradis et al., 2009; Alexander et al., 2013) Interestingly, pathologically activated STAT1 signaling promotes inflammation and fibrosis in high-fat diet fed mice, while enhanced IL-6-STAT3 signaling is instrumental in eliciting tumor development, reaffirming previous findings describing an essential role for the IL-6-STAT3 axis in obesity-related HCC. (Park et al., 2010; Grohmann et al., 2018) Trans-signaling of IL-6 has been linked to the expansion of progenitor cells in mouse models of chronic injury, thereby favoring the development of combined hepatocellular-cholangiocellular carcinomas. (Rosenberg et al., 2022) Although an autocrine loop for IL-6/STAT3 in hepatocytes has been suggested, activated KCs and infiltrating macrophages are also a vital source of IL-6 and TNF-α, which is also elevated in NAFLD/NASH mouse models. (Park et al., 2010; Yu et al., 2019) Alterations in lipid composition and metabolism are a common feature in primary liver cancer and promote tumor growth and progression. (Satriano et al., 2019) Lipids are essential for the synthesis of cell membranes and other structures, but are also stored and used as energy source, serving as substrate for fatty acid oxidation (FAO) in mitochondria. Importantly, lipids also act as signaling molecules and substrates for bioactive molecules. (Paul et al., 2022) Although not utilizing lipids for FAO, malignant liver cells have an increased demand for lipids to build cell membranes and mediators that promote tumor progression. (Paul et al., 2022) Subsequently, genes included in FFA uptake and in particular *de novo* lipogenesis are upregulated in HCC. (Luo et al., 2021) As a result, the HCC TME is enriched in fatty acids and lipid derivatives, an environment that can promote a pro-tumoral phenotype in tumor-associated macrophages (TAM), characterized by the expression of CD206, IL-6, vascular endothelial growth factor A (VEGF-A), matrix metalloproteinase (MMP) 9 and ARG1. (Wu et al., 2019a; Broadfield et al., 2021) Lipid-binding nuclear receptors such as peroxisome proliferator activated receptors (PPAR) and liver X receptor (LXR) are important regulators of macrophage metabolism and polarization. (Odegaard et al., 2007; Pourcet et al., 2016) PPAR-γ has a particularly decisive role for TAM metabolism. PPAR-γ activity induces FAO in macrophages and upregulates CD36 expression. (Nagy et al., 1998; Odegaard et al., 2007) On the other hand, cleavage of PPAR-γ by caspase 1 allows its binding to the mitochondrial protein medium-chain acyl-CoA dehydrogenase (MCAD) resulting in reduced FAO and increased production of lactate, indicative of glycolytic activity. (Niu et al., 2017; Wu et al., 2020; Bogdanov et al., 2022) LXR, a nuclear receptor involved in cholesterol regulation, was suggested to contribute to the phenotype of TREM2^+^ macrophages in HCC. (Zhou et al., 2022) Interestingly, the transcriptomic profile of TREM2^+^ macrophages in NASH liver closely resembles TREM2^+^ TAMs found in HCC, in both patients and mouse models. (Zhang et al., 2022; Zhou et al., 2022) Even more intriguingly, gene signatures of human TREM2^+^ macrophages are also reminiscent of those from murine TREM2^+^ macrophages, suggesting a highly conserved (and thus pivotal) role for this macrophage population. (Mulder et al., 2021)

#### 4.1.2 The hypoxic and acidic tumor environment

Tumor cells acquire a drastically deregulated metabolism, which is not only an adaptation to an unfavorable environment often characterized by hypoxia and nutrient deprivation, but also affects the metabolism and phenotype of macrophages and impairs their immune competence. Tumor cells actively contribute to generating an acidic microenvironment by aerobic glycolysis, notably through the generation of lactate. (Hanahan and Weinberg, 2011; Bogdanov et al., 2022) This acidic environment induces the immunosuppressive protein cyclic AMP element modulator (CREM) and enables binding of the myeloid-derived acidic pH selective V-domain immunoglobulin suppressor of T cell activation (VISTA) to its receptor on T cells, suppressing cytotoxicity. (Sawka-Verhelle et al., 2004; Lines et al., 2014; Johnston et al., 2019) In addition, aerobic glycolysis reduces glucose availability for macrophages, thus limiting one of the metabolic pathways leading to the generation of reactive oxygen species (ROS) and the release of inflammatory cytokines. (Freemerman et al., 2014) Increased lactate concentrations further stabilize hypoxia-inducible factor (HIF)-1α, thereby driving a tumor-promoting phenotype in bone marrow-derived, tumor-educated TAMs, characterized by enhanced expression of arginase and VEGF. (Colegio et al., 2014) Hypoxia resulting from insufficient oxygen supply, is a typical phenomenon in acute and chronically injured tissue and is a critical inducer of regenerative processes, primarily orchestrated by the major hypoxia sensing transcription factor HIF-1α. (Ruthenborg et al., 2014) These wound healing processes are also abundant but dysregulated in tumors, where hypoxia is a result of an inadequate oxygenation due to rapid tumor cell proliferation and defective vascularization. (Byun and Gardner, 2013; Lv et al., 2017; Abou Khouzam et al., 2020) Macrophages play a critical role in both physiological and pathological wound healing and their functional activation is influenced by hypoxia. Indeed, HIF-1α signaling was shown to upregulate PD-L1 expression in murine splenic and tumor-associated myeloid cells (MDSCs). (Noman et al., 2014) Accordingly, CD8^+^ T cells from highly hypoxic HCC tissue areas displayed an increased expression of PD-1 along with a decreased expression of Granzyme B, compared to CD8^+^ T cells from tumor regions with low hypoxia, indicative of an anergic, less cytotoxic phenotype. (Suthen et al., 2022) Using a murine model of orthotopic HCC, another study demonstrated that HIF-1α not only induces PD-L1, but also promotes Triggering receptor expressed on myeloid cells 1 (TREM-1) expression on TAMs. Strikingly, TREM-1 signaling upregulated CCL20 expression in TAMs, resulting in recruitment of CCR6^+^ Tregs to hypoxic areas and thus participating in tumor resistance against PD-L1 blockade. (Wu et al., 2019b)

HIF-1α signaling in TAMs induces not only numerous genes involved in epithelial-mesenchymal transition (EMT), immunosuppression and regeneration, but also genes involved in glucose metabolism, such as Glucose transporter 1 (GLUT1) and hexokinase 2 (HK2), shifting TAM metabolism towards aerobic glycolysis further amplifying this phenomenon and maintaining a TAM anti-inflammatory phenotype. (Puthenveetil and Dubey, 2020; de-Brito et al., 2020)

### 4.2 The diverse origins and roles of tumor-associated macrophages in the tumor microenvironment

#### 4.2.1 Defining the tumor-associated macrophages

As illustrated by their diverse roles in immune homeostasis and tissue injury as well as in repair mechanisms, macrophages are equipped with a plethora of anti-inflammatory and pro-regenerative properties, which can be exploited by a tumor. The predominant, detrimental role of TAMs is to protect the malignancy from the host anti-tumor immunity. However, TAMs are also involved in the formation of new blood vessels, the supply with growth factors, the support of epithelial to mesenchymal transition and tumor cell dissemination, as well as resistance towards chemotherapies, which are considered important hallmarks of cancer progression. (Hanahan and Weinberg, 2011; Hanahan, 2022)

While the accumulation of oncogenic mutations and the attainment of proliferative independency is the basis of carcinogenesis, the tumor stroma provides a critical supportive and protective niche for the developing tumor. The TME comprises non-malignant acellular and cellular components such as secreted factors, ECM as well as cancer associated fibroblasts, endothelial cells, and heterogeneous innate and adaptive immune cells. (Hanahan and Weinberg, 2011; Anderson and Simon, 2020) The composition of the TME varies greatly depending on the tumor type, underlying mutations, tumor stage and metabolic conditions within the tumor. (Hanahan and Weinberg, 2011; Anderson and Simon, 2020; Llovet et al., 2021) The immune compartment of the TME contains various immune cells that either actively support the tumor (for instance, macrophages, neutrophils, regulatory T cells (Treg), regulatory B cells) or that are potentially tumoricidal but rendered ineffective by the anti-inflammatory environment (such as macrophages, T cells, dendritic cells, NK cells, NKT cells). (Binnewies et al., 2018) Besides Treg cells, myeloid cells represent a major immunosuppressive compartment in the tumor stroma, and are composed of granulocytes, TAMs and immature myeloid cells, often referred to as myeloid-derived suppressor cells (MDSCs). (Binnewies et al., 2018) Monocytes/MDSCs and TAMs constitute a major part of the tumor stroma in solid tumors and are often indicative of a poor prognosis.

Monocytic MDSCs (M-MDSCs) are described as CD11b^+^Gr1/Ly6C^high^Ly6G^−^ cells in mouse and CD11b^+^CD14^+^HLA-DR^−/lo^CD15^-^ cells in humans. Granulocytic or polymorphonuclear MDSCs (PMN-MDSCs) are defined as CD11b^+^Gr1/Ly6C^low^Ly6G^+^ in mouse, while human PMN-MDSCs are mostly described as CD11b^+^CD14^−^CD66b^+^CD15^+^. However, as MDSCs express identical surface markers as classical monocytes and neutrophils, functional assays or additional markers are needed to identify MDSCs. Moreover, the use of the term MDSC is disputed in the literature and many studies do not clearly distinguish between TAMs, classical monocytes or monocytic MDSCs. While some authors advertise MDSCs as a coequal immune cell population, other researchers regard the concept of MDSCs as oversimplified and outdated. (Bronte et al., 2016; Guilliams et al., 2018; Veglia et al., 2018)

Accumulation of macrophages in patients with HCC was reported to be generally associated with disease progression or aggressiveness. (Ding et al., 2009; Yeung et al., 2015) Although the majority of TAM exert anti-inflammatory and tumor supporting functions, macrophages also have the inherent ability to initiate an anti-tumor response and eliminate malignant cells directly by phagocytosis or indirectly by eliciting a Th1 response. (Martinez et al., 2008; Mantovani et al., 2017) In a well-noticed study of macrophages in patients with colon cancer, a high density of TAMs correlated with a better clinical outcome. (Forssell et al., 2007) Because of the dual roles of TAM and the observation that tumor-promoting macrophages express phenotypic markers resembling to some extent the so-called M2-macrophages, while anti-tumoral macrophages share functional markers with the so-called M1-polarized macrophages, TAMs were classified according to the M1/M2 dichotomy. However, over the last years it became evident, that TAMs and liver macrophages in general are phenotypically and functionally much more heterogeneous than previously assumed, and that distinct phenotypes may even coexist within a singular tumor, thus the M1/M2 paradigm is too simplified and should not be applied anymore. (Cassetta et al., 2019; Donadon et al., 2020) Still, in some human studies, TAMs with an inflammatory phenotype are referred to as M1-like TAMs or TAM1, whereas TAMs with tumor-promoting properties are described as M2-like TAMs or TAM2. Tumoricidal TAM1 are mostly identified by immunogenic markers like CD68 and CD80, CD86, MHC class II or iNOS. (Movahedi et al., 2010) Tumor-promoting TAM2 are generally defined by the expression of scavenger receptors (e.g., CD204, CD206, CD163 or CD169) or ARG1. (Christofides et al., 2022) However, this nomenclature ignores that some of the inflammatory “M1” characteristics, like iNOS or TNF-α production can also fuel cancer progression. (Greten and Grivennikov, 2019)

In a recent cross-tissue meta-analysis of human macrophage single-cell datasets, Mulder et al. identified at least four distinct TAM populations in HCC, one of them being uniquely found in liver cancer, while another population of inflammatory IL-1β^+^ monocytes present in other cancer entities was not accumulating in HCC. (Mulder et al., 2021) In particular, they identified and described a subset of IL-4I1^+^ TAMs conserved across all cancer entities including lymph node metastasis. While IL-4I1^+^ TAMs were characterized by increased levels of the immunosuppressive markers IDO and PD-L1, they also expressed immunostimulatory “M1”-like markers, such as HLA-DR, CD86 as well as interferon (IFN)-yR and CD40, indicative of an interactions with T cells. The authors demonstrated that interactions with CD40L^+^ CD4 T cells together with CD8^+^ T cell derived IFN-y programmed the IL-4I1^+^ TAMs towards an immunosuppressive phenotype. Another TAM subset, characterized by expression of *TREM2* and *SPP1* resembling recently identified fibrosis associated macrophages, was present in all primary tumors but not in metastasis. (Mulder et al., 2021) A further subpopulation of TAMs was found to express the Notch target HES1 and high amounts of CD206 and CD163 and to interact preferentially with Tregs. (Sharma et al., 2020; Mulder et al., 2021) Interestingly, HES1^+^ TAMs in HCC express the folate 2 receptor *FOLR2* and resemble fetal liver macrophages. (Sharma et al., 2020) TAM subpopulations seemingly not only differ in gene expression signature but also in their localization in the TME. HES1^+^ TAMs and TREM2^+^ TAMs were preferentially located in the tumor tissue while IL-4I1^+^ TAMs were enriched at the tumor periphery. (Mulder et al., 2021)

The M1/M2 and MDSC concept is controversial, oversimplified and in many aspects inaccurate in regards of TAM subpopulations, therefore we will not refer to this nomenclature in the following section but rather describe the functional and phenotypical features of macrophages and monocytes in the TME.

#### 4.2.2 The origin of TAMs in liver cancer

Although alternative routes exist, CCL2 is considered as the major chemokine responsible for the recruitment of classical/pro-inflammatory monocytes in manifold inflammatory conditions. In the diseased liver, CCL2 is expressed by activated HSCs and fibroblasts, activated KCs, endothelial cells, injured biliary epithelial cells, and premalignant hepatocytes. (Eggert et al., 2016; Ehling and Tacke, 2016; Guillot et al., 2021) However, malignant cells frequently overexpress CCL2 as well, and high expression of CCL2 in human HCC correlates with a poor prognosis. (Li et al., 2017a)

In a model of Hepa 1-6 cell-derived liver tumors, developed in otherwise unchallenged mouse livers, CCL2/CCR2 blockade attenuated tumor growth, altered TAM phenotype and restored CD8^+^ T cell activity. (Li et al., 2017a) Similarly, in a model of DEN + CCl_4_-induced fibrosis-HCC, disruption of the CCL2/CCR2 axis reduced tumor burden and pathological vascularization. (Bartneck et al., 2019) The role of TAM recruitment in NAFLD/NASH associated HCC on the other hand is yet unclear. In mouse models of NASH-HCC, CCR2 deficiency did not ameliorate tumor development suggesting alternative sources of TAMs or alternative recruitment pathways involved in fatty liver disease progression. (Wolf et al., 2014)

Indeed, besides CCL2, there are many other chemokines and cytokines involved in the recruitment and accumulation of monocytes/MDSCs and TAMs at the tumor site. For example, CCL5 and CCL3, binding to their cognate receptors CCR1 and CCR5 on monocytes, have been shown to attract tumor promoting myeloid cells, supporting HCC progression and metastasis, respectively. (Ehling and Tacke, 2016) Additionally, cytokines such as M-CSF (CSF-1) and VEGF-A have been implicated in the recruitment and differentiation of TAMs in murine HCC models. (Zhu et al., 2019; He et al., 2021) Also, sustained release of inflammatory mediators such as GM-CSF, CXCL12, and G-CSF by chronic inflammation in tumors causes emergency myelopoiesis, an enhanced expansion of the myeloid niche in bone marrow and spleen, and the release of immature myeloid cells with an immunosuppressive phenotype into the circulation, potentially giving rise to TAMs. (Arvanitakis et al., 2022)

Although TAMs are mostly derived from circulating CCR2^+^ monocytes, it is known that they can proliferate within the tumor site and that tissue resident macrophages (i.e. KCs) can contribute to the TAM pool, as well. (Mantovani et al., 2022) For example, local proliferation of TAMs in human HCC was linked to tumor-derived adenosine, acting synergistically with autocrine GM-CSF. (Wang et al., 2021) Also, binding of CSF-1 to its receptor CD115 on macrophages is known to mediate macrophage survival and proliferation. (Yu et al., 2012; Krenkel and Tacke, 2017)

Using RNA velocity, recent scRNAseq studies revealed that a TAM population with an embryonic signature in human liver cancer, characterized by high expression of HES1, FOLR2, CD163 and CD206 was at least partially derived from tissue resident macrophages, while other TAM subsets (TREM2^+^ TAMs and IL-4I1^+^ TAMs) were identified to be monocyte-derived. These results were confirmed using an elegant fate-mapping mouse model based on Ms4a3^Cre^-Rosa^TdT^ reporter mice. (Sharma et al., 2020; Mulder et al., 2021) Interestingly, the aforementioned HES1^+^ TAM population was mainly located within the tumor, while the monocyte-derived TAM subpopulations rather accumulated in the tumor periphery, which is in good agreement with previous observations that CCR2^+^ TAMs with an inflammatory and pro-angiogenic gene signature accumulated at the tumor border, while CD163^+^ TAMs were predominantly found in the tumor center. (Bartneck et al., 2019; Mulder et al., 2021)

#### 4.2.3 Phagocytosis and scavenging—the role of anti-inflammatory markers

Removal of unwanted and dead material is a central function of macrophages and can be immunogenic in case of tissue damage and inflammation and is then called phagocytosis, or non-immunogenic to preserve tissue homeostasis, a process termed efferocytosis. Phagocytosis of tumor cells and debris by macrophages not only eliminates tumor cells but induces cytokine production and cross-presentation of tumor antigens to CD8^+^-T cells, hence holding an important role in tumor control. (Biswas and Mantovani, 2010) The phagocytic activity of macrophages is tightly regulated by the balance between “eat me” and “do not eat me”-ligands on cells that are screened by patrolling macrophages. (Lecoultre et al., 2020) Typical “eat me” signals that induce phagocytosis include opsonizing antibodies binding to FcyR on TAMs and the exposure of calreticulin on the surface of cancer cells. (Obeid et al., 2007; Cassetta et al., 2019) “Do not eat me” ligands, like CD47 and PD-L1, are frequently upregulated in tumors and protect the malignant cells from phagocytic elimination. (Lecoultre et al., 2020) Binding of the ubiquitously expressed molecule CD47 to its receptor signal-regulatory protein alpha (SIRPα) on TAMs, effectively inhibits phagocytosis. CD47 is frequently increased in HCC and strongly overexpressed in cholangiocarcinoma, where blockade of CD47/SIRP1a interaction enhanced phagocytosis and reduced tumor progression. (Xiao et al., 2015; Chen et al., 2019a; Vaeteewoottacharn et al., 2019) Macrophage-driven cell clearance (efferocytosis/phagocytosis) has gained increasing attention in NAFLD/NASH. A recent study demonstrated that the prolonged hypernutrition in fatty livers leads to the impairment of TREM2-dependent macrophage efferocytic activity, thereby exacerbating liver inflammation and NASH progression. (Wang et al., 2023) Additionally, another finding indicated that the CD47-SIRPα axis blockade can reverse the inhibition of macrophage-driven cell clearance, and decrease liver fibrosis. (Shi et al., 2022) Neoplastic cells in HCC can express PD-L1, which was shown to impede T cell activity. (Calderaro et al., 2016) Of note, murine and human TAMs were reported to express PD-1. PD-1^+^ TAMs displayed a reduced phagocytic activity against PD-L1^+^ tumor cells, which could be restored in mouse models of cancer by blocking the PD-1/PD-L1 axis. (Gordon et al., 2017) While reinforcing phagocytosis is an interesting therapeutic target, phagocytosis is a process intimately linked to inflammation resolution and might promote macrophages to differentiate towards an anti-inflammatory phenotype. (Wang et al., 2014) Apoptotic cell death is characterized by the exposure of the “eat me” signal phosphatidylserine on the surface of the dying cells. Phosphatidylserine is recognized by various engulfment receptors of the “TAM family” (Tyro3, Axl and Mer) on macrophages and induces non-immunogenic phagocytosis termed efferocytosis. (Lecoultre et al., 2020) Unlike phagocytosis, efferocytosis does not induce antigen cross-presentation but mediates an anti-inflammatory effect and has been implicated in tumor support. (Lecoultre et al., 2020) However, while overexpression of the efferocytosis receptor MerTK on human HCC cells was linked to tumor growth, there is little data available about the role of efferocytosis in TAMs for primary liver cancer. (Liu et al., 2022)

Similarly, removal of potentially harmful or excess molecules by scavenger receptors is a crucial function of macrophages in homeostasis and mostly assigned to an anti-inflammatory macrophage phenotype. Scavenger receptors are a diverse group of membrane-bound receptors that recognize and internalize a wide range of exogenous and endogenous ligands. The scavenger receptors CD206, CD163 and CD204 are upregulated on pro-tumoral TAMs in HCC and correlate with a poor prognosis. (Yeung et al., 2015; Li et al., 2017b; Ding et al., 2019; Mulder et al., 2021) Although CD204, CD206 and CD163 are commonly used as markers for tumor-promoting TAM, the exact role of these receptors in tumor promotion is not well understood. A high intra-tumoral density of CD204, also named scavenger receptor A 1 (SR-A1) or macrophage scavenging receptor 1 (MSR-1), is associated with a poor overall survival of patients with HCC. (Ding et al., 2019) Interestingly, CD204 can signal through MerTK to facilitate uptake of apoptotic material, a typical trait of a tolerogenic macrophage phenotype. (Todt et al., 2008; Lecoultre et al., 2020) On the other hand, Guo et al. demonstrated that *in vitro* engagement of CD204 resulted in JNK-induced upregulation of inflammatory genes, like *Tnfa, Il1b* and *Ccl2,* in IL-4 primed alternatively activated murine macrophages, suggesting a role in macrophage polarization. (Guo et al., 2019) The mannose receptor CD206 binds a wide array of pathogen-derived fragments and endogenous ligands including tumor-derived mucins, and is widely used as a phenotypical marker for anti-inflammatory macrophages and TAMs, both in human and mice. Emphasizing its immunosuppressive nature, CD206 is mainly found on immunoregulatory cells and triggering of CD206 promotes IL-10 production and induces a tolerogenic phenotype in activated CD8^+^ T cells. (Allavena et al., 2010; van der Zande et al., 2021) Contrarily, in models of melanoma and colon cancer high CD206 expression on TAMs was associated with improved cross presentation of tumor neoantigens to CD8^+^ T cells resulting in better tumor control. (Modak et al., 2022) The expression of the scavenger receptor CD163 is restricted to macrophages and to a lesser extend monocytes and closely linked to an anti-inflammatory phenotype. (Skytthe et al., 2020) CD163 binds haptoglobin-hemoglobin complexes and removes toxic free hemoglobin and damaged erythrocytes from the circulation thereby stimulating the production of anti-inflammatory heme-metabolites. (Canton et al., 2013; Skytthe et al., 2020) Sharma et al. demonstrated that high expression of CD206 and CD163 is a hallmark of TAMs with an onco-fetal-like phenotype in human HCC, which can be induced by tumor-associated endothelial cells through DLL4-NOTCH signaling. These TAMs showed increased interactions with immunosuppressive Tregs and expressed the angiogenic factor CXCL12. (Sharma et al., 2020) As mentioned above, CD36 upregulation and lipid accumulation has also been described on pro-tumoral TAMs and infiltrating CD11b^+^ Ly6C(Gr1)^+^ myeloid cells from different cancer entities in mice. TAMs thus engage in fatty acid oxidation to generate energy, a metabolic profile associated with immunosuppressive characteristics. (Al-Khami et al., 2017; Puthenveetil and Dubey, 2020; Su et al., 2020) In mouse models of liver metastasis, Yang et al. demonstrated that tumor cells release fatty acid-loaded vesicles that are captured and internalized through the lipid transporter CD36 on CD206^+^ metastasis associated macrophages (MAMs). MAMs as well as bone marrow-derived TAMs, co-cultured with different tumor cell lines, displayed an increased capability to take up long chain fatty acids. Of note, uptake and accumulation of fatty acids induced macrophage polarization towards an immunosuppressive phenotype. (Yang et al., 2022)

#### 4.2.4 TAMs directly fuel a tumor-promoting inflammatory response

Tumor-promoting inflammation is an enabling hallmark of cancer and primary liver cancers are a typical example of inflamed tumors. Inflammatory processes are involved in tumor initiation, promotion and progression. Tumor-promoting inflammation includes not only the recruitment of immune cells by the tumor and TME-derived chemokines but also the release of inflammatory cytokines that support tumor growth. During tumor initiation, premalignant cells require an inflammatory environment to undergo oncogenic transformation and gain additional mutations. While inflammatory mediators produced by premalignant cells can act in an autocrine manner, the released chemokines activate tissue resident macrophages and attract monocytes and granulocytes from the circulation. Recruited myeloid cells produce ROS and reactive nitrogen species further driving mutations in the tumor cells but at the same time produce inflammatory cytokines that activate pro-survival pathways in tumor cells. (Greten and Grivennikov, 2019)

Tumor initiation in the liver is largely dependent on pro-inflammatory activated hepatic macrophages, as demonstrated in murine models. During chronic liver inflammation, oxidative stress and the associated ROS accumulation in hepatocytes induce the release of factors that activate liver macrophages. (Yuan et al., 2017) The IL-6/STAT3 and TNF-α/NF-κB pathways are key drivers of hepatocarcinogenesis. In particular, obesity-promoted HCC initiation depends on increased levels of IL-6 and TNF-α. (Park et al., 2010) In a murine model of endoplasmic reticulum stress and steatosis, macrophage-derived TNF promoted aggravation of steatohepatitis and HCC development. (Nakagawa et al., 2014) TNF-α not only facilitates tumor initiation but it also promotes tumor cell survival and proliferation *via* NF-κB activation. (Luo et al., 2004; Nakagawa et al., 2014; Yuan et al., 2017) IL-6 is a multifunctional NF-κB-regulated cytokine that activates the STAT3 pathway and promotes hepatocyte survival and proliferation. (Campana et al., 2018) Using hepatoma cell lines, Chen et al. demonstrated that IL-6 secretion by TAMs derived from primary human monocytes can induce upregulation of CD47 on tumor cells, protecting the tumor from phagocytosis and thus enhancing tumor development. During tumor progression, inflammation triggers cancer cell stemness *via* STAT3 dependent pathways. (Greten and Grivennikov, 2019) Cancer stem cells are a small subpopulation of dedifferentiated or stem-like tumor cells, with a high potency for self-renewal, contributing to therapy resistance, cancer relapse and intratumoral cell heterogeneity. (Huang et al., 2020; Walcher et al., 2020)

TAMs can directly facilitate HCC growth by the secretion of growth factors, such as HGF, epithelial growth factor receptor (EGFR)-ligands, fibroblast growth factor (FGF), platelet-derived growth factor PDGF, insulin-like growth factor-1 (IGF-1) and TGF-β. *In vitro*, HGF and IGF secreted by CD163^+^ macrophages were sufficient to induce hepatoma cell proliferation. (Sprinzl et al., 2015; Dong et al., 2019) TGF-β is frequently overexpressed in the TME of HCC where it promotes de-differentiation and metastasis, but also a tumor-supporting phenotype of TAMs. (Dituri et al., 2019) RAW264.7 derived TAMs displayed an elevated expression of TGF-β in comparison to other macrophage phenotypes, highlighting their participation in these processes. (Fan et al., 2014)

TREM2 is highly expressed in TAMs in over 200 different human tumors but is absent or weakly expressed in most healthy tissues. (Molgora et al., 2020) In murine tumor models, genetic ablation of TREM2 decreased the number of intra-tumoral immunosuppressive TAMs and noticeably improved the efficacy of an anti-PD-1 treatment. (Katzenelenbogen et al., 2020; Molgora et al., 2020; Binnewies et al., 2021) Furthermore, TREM2^+^ TAMs were described to co-express various factors that facilitate tumor immune evasion, such as *Arg1*, *Gpnmb* or *Spp1*. (Sharma et al., 2020; Lazaratos et al., 2022) Likewise, anti-inflammatory TREM2^+^ TAMs were reported in primary liver cancers of patients and in mouse models. (Zhang et al., 2019a; Mulder et al., 2021; Zhou et al., 2022).

Interestingly, in a scRNAseq study of murine NASH livers, proliferating macrophages were predominantly co-expressing *Trem2*, *Gpnmb* and *Spp1*. (Zhang et al., 2022) *Spp1* encodes the protein osteopontin (OPN), which is also involved in tumor promotion and predicts a poor outcome in HCC. OPN induces expression of CSF1, and a global *Spp1* knock-out decreased numbers of TAMs and suppressive monocytes/MDSCs suggesting a role for TREM2^+^ TAMs in macrophage survival and proliferation. (Zhu et al., 2019)

#### 4.2.5 Immunosuppression/interaction with T cells

The host’s immune surveillance is one of the major obstacles for a developing malignancy. Cytotoxic T cells and NK cells are highly efficient in killing tumor cells and are supported by anti-tumoral macrophages that phagocytose tumor cells and cross-present tumor-antigens to T cells. (Martinez-Lostao et al., 2015; Lecoultre et al., 2020) In the early phase of HCC development, macrophages act as sentinels and are indispensable for the CD4^+^ T cell mediated clearance of premalignant hepatocytes. (Kang et al., 2011) Premalignant hepatocytes can enter a state of growth arrest termed oncogene-induced senescence, a stress response to aberrant function of oncogenes, in which they acquire a secretory phenotype and release inflammatory cytokines and chemokines, amongst others CCL2 to recruit immune cells and initiate their own removal. However, once a tumor succeeds in growing and establishes an inflammatory TME, monocyte-derived macrophages recruited *via* the CCL2/CCR2 axis are rapidly reprogrammed and protect the growing tumor by NK cell inactivation. (Eggert et al., 2016)

Immunosuppression is a crucial mechanism to prevent an overshooting immune response resulting in auto-aggression. TAMs produce the anti-inflammatory cytokine IL-10, that induces and promotes regulatory T cells and efficiently inhibits the differentiation of cytotoxic T cells as well as the expression of inflammatory cytokines. (Mannino et al., 2015) In addition, TAMs can release chemokines, such as CXCL9, 10, and 11, and CCL22 that attract Tregs through CXCR3 and CCR4, respectively. (Ehling and Tacke, 2016; Mulder et al., 2021) Furthermore, strong evidence from imaging mass cytometry in human melanoma, suggest that CXCL9 is a major chemoattractant for cytotoxic CD8^+^ T cells, and CXCL9 and CXCL10 expressing cells accumulating in areas of active anti-tumor activity. (Hoch et al., 2022) Similar phenotypes in liver cancer are yet to be demonstrated.

Immunosuppressive myeloid cells can inhibit cytotoxic T cells either directly by cell-cell-interactions or by withdrawing essential metabolites needed for cytotoxic activity. For instance, TAMs express high amounts of ARG1, an enzyme that catabolizes arginine, a critical amino acid needed for T cell activation. By removing arginine from the microenvironment, TAMs effectively inhibit the T cell-driven anti-tumor response. Similarly, the expression of the enzymes indoleamine 2,3-dioxygenase 1 (IDO1) and interleukin-4-induced 1 (IL-4I1) are increased in a subpopulation of both human and murine TAMs in HCC. (Mulder et al., 2021) IDO1 and IL-4I1 are involved in the enzymatic conversion of tryptophan to kynurenin and other metabolites, which engage the aryl hydrocarbon receptor (AhR). AhR activation facilitates immune tolerance in a variety of immune cells, especially T cells, by promoting Treg differentiation and upregulating PD-1 on cytotoxic T cells. (Mezrich et al., 2010; Liu et al., 2018; Sadik et al., 2020) Thus, in TAMs, AhR activation induces the expression of IL-6, which in turn enhances the activity of IDO1. (Cheong and Sun, 2018) Another well-known mechanism of tumor escape involves the PD-1/PD-L1 axis. Noteworthy, similarly to cancer cells, TAMs highly express the immune checkpoint ligand PD-L1. Binding of those molecules to PD-1 on activated T cells attenuates T cell cytotoxic activity. (Kuang et al., 2009) Likewise, PD-L1^+^ TAMs are primarily found in the peritumoral stroma of HCC patients, where cytotoxic T cells accumulate. (Kuang et al., 2009; Mulder et al., 2021) Another inhibitory checkpoint ligand expressed by macrophages is VISTA, which interacts with its receptor PSGL1 on T cells and inhibits their proliferation and cytokine production, while inducing Foxp3 and favoring Treg function. (Lines et al., 2014; Johnston et al., 2019).

Although many cells within the TME produce cytokines, TAMs are considered as major source of tumor-promoting NF-κB-regulated cytokines, such as IL-6, IL-23, IL-1β, IL-10, TNF-α and TGF-β, that can activate oncogenic pathways like STAT3 in cancer cells but also participate in the establishment of a pro-tumoral microenvironment. (Greten and Grivennikov, 2019) Although IL-10 is produced by a variety of cells, TAMs are a significant source of IL-10 in the TME. Increased levels of IL-10 in human HCC are associated with poor survival. (Zhang et al., 2019b) For instance, IL-10 inhibits the expression of MHC-II and inflammatory cytokines and was shown to induce PD-L1 expression on macrophages, an effect that is potentiated by TNF-α and prostaglandin E2 (PGE_2_), also produced by TAMs. (Kuang et al., 2009; Pu and Ji, 2022) Moreover, CCL22 and CCL17 secreted by TAMs attract Th2 T cells and induce Th2 polarization of naive T helper cells. (Gieseck et al., 2018) Th2 T cells are ineffective against tumors but produce cytokines like IL-4 and IL-13 that promote wound healing and fibrosis and skew macrophages towards a restorative, pro-tumoral phenotype. (Balkwill, 2004; Barron and Wynn, 2011) Bacterial components, as typically observed in the liver in pre-neoplastic conditions such as NAFLD, also participate in tumor promoting inflammation. IL-23, a pro-inflammatory cytokine, is secreted by macrophages in response to lipopolysaccharide exposure. (Peral de Castro et al., 2012) IL-23 induces Th17 polarization of T cells, recruitment of neutrophils and activation of DCs and TAMs. While in colorectal cancer, IL-23 aggravated tumor progression, there is only little evidence for a crucial role of TAM-derived IL-23 in HCC. (Grivennikov et al., 2012; Heredia et al., 2022) However, as shown in *in vitro* studies and an ectopic tumor model using immunodeficient mice TAM-driven Th17 lymphocyte activation participates in HCC progression. (Gasmi et al., 2022) IL-1β, a prototypic pro-inflammatory cytokine, is involved in metabolic liver diseases and is activated by caspase-1 *via* the NLRP3 inflammasome. (Knorr et al., 2020) Paradoxically, IL-1β promotes tumor immune evasion and tumor progression. IL-1β produced by inflammatory macrophages increases PD-L1, as well as HIF-1α-dependent CSF-1 expression in hepatoma cells, facilitating TAM accumulation. (Zong et al., 2019; He et al., 2021)

#### 4.2.6 The roles of macrophages in angiogenesis

To maintain their growth and survival, tumor cells need nutrients and oxygen, normally provided by the hosts blood system. Insufficient oxygen supply by blood vessels due to the rapid growth, high nutrient demand and sheer mass of tumor cells results in hypoxia, a state of pathologically low levels of oxygen. (Abou Khouzam et al., 2020) Hypoxia is a common phenomenon in malignant tumors. It is predominantly sensed by the transcription factor HIF-1α in malignant and stromal cells and induces a plethora of target genes that promote reprogramming of tumor and TME metabolism, as well as angiogenesis to adapt to the hypoxic environment. Important angiogenic factors induced by hypoxia are angiopoietin 2 (Ang2), CXCL12 (also known as SDF-1) and VEGF-A. (Abou Khouzam et al., 2020) The key role of TAMs in angiogenesis is well-established and removing proangiogenic TAMs abrogates tumor vascularization, and impairs tumor growth in murine tumor models. (Zhang et al., 2010; Bartneck et al., 2019) Indeed, TAMs accumulate in hypoxic areas and express several pro-angiogenic factors, such as VEGF-A, CCL18 and MMP9. (Song et al., 2020; Lu et al., 2022)

In 2005, De Palma et al. identified a subset of pro-angiogenic monocytes expressing Tie2, a receptor binding Ang2 that gave rise to pro-angiogenic TAMs. (De Palma et al., 2005) HCC is a hypervascular tumor characterized by elevated levels of Ang2. (Mitsuhashi et al., 2003) In line with this, Tie2^+^ circulating monocytes and Tie2^+^ liver macrophages were increased in HCC patients and microvessel density in human HCC correlated with frequency of pro-angiogenic Tie2^+^ macrophages. (Matsubara et al., 2013) Tie2^+^ expressing pro-angiogenic monocytes and macrophages express high levels of the CXCL12 receptor CXCR4, and the anti-inflammatory macrophage marker CD206, MMP9 and VEGF-A. (Coffelt et al., 2010) CCR2^+^ monocyte-derived macrophages particularly favor pathogenic angiogenesis (“arterialization”) in mouse models of primary HCC in a fibrotic environment. (Bartneck et al., 2019)

Pro-angiogenic TAM were reported to promote resistance to chemotherapy and vascular-disrupting therapies as they are recruited through CXCR4 to CXCL12-enriched hypoxic regions of tumors where they stimulate revascularization. (Welford et al., 2011; Nakasone et al., 2012) In a scRNA-seq study including samples from seven viral hepatitis related human HCCs, Song et al. described a subset of TREM2^+^ CD206^+^ TAMs expressing CCL18, a chemokine involved in angiogenesis. (Lin et al., 2015; Song et al., 2020) Interestingly, this TAM cluster displayed a pathway activity enriched for hypoxia, iron transport and lipid metabolism and was enriched in CREM (also known as ICER), a protein induced by tumor acidosis that inhibits TLR-dependent NF-κB signaling. (Sawka-Verhelle et al., 2004; Harzenetter et al., 2007; Song et al., 2020)

The tumor endothelium in HCC is characterized by a high expression of CXCR4, a marker for neoangiogenesis. (Xu et al., 2017) Meng et al. demonstrated that TAM derived TNF-α and to a lesser extend other pro-inflammatory cytokines promoted CXCR4 expression on tumor endothelium, which suggests that TAMs induce a pro-angiogenic phenotype in tumor-associated endothelial cells. (Meng et al., 2018)

#### 4.2.7 Macrophages regulate the dissemination of cancer cells

Invasive growth and dissemination characterize aggressive tumors, and metastatic cancer causes approximately 90% of cancer-related death. (Ganesh and Massague, 2021) Epithelial to mesenchymal transition (EMT) is a crucial mechanism during development and tissue regeneration but it is also the initial step of metastasis. During EMT, epithelial cells lose their typical epithelial expression and downregulate adhesion proteins like E-cadherin to detach from neighboring cells. At the same time, they upregulate mesenchymal markers like vimentin and acquire an invasive, migratory phenotype. Important transcription factors regulating EMT are Snail, Slug and Twist. (Yan et al., 2018; Li et al., 2022) Similarly to what was observed during tissue regeneration, pathological EMT in tumors is regulated by macrophages. TAMs accumulate at the invasive borders of the tumor and secrete growth factors, cytokines and MMPs, that in concert facilitate EMT and metastasis of tumor cells. One of the most powerful EMT inducing factors produced by TAMs is TGF-β, which has a dual role in liver cancer. While TGF-β is known to inhibit proliferation of mature hepatocytes and to suppress early stages of tumorigenesis, it facilitates cancer stemness and metastasis in advanced tumors *via* the YAP/TAZ pathway and Snail. (Fan et al., 2014; Moon et al., 2017; Yan et al., 2018) TAMs can secrete TGF-β directly but can also activate latent TGF-β in the ECM by releasing serine proteases and MMPs (e.g., MMP2, MMP9). (Kessenbrock et al., 2010; Fan et al., 2014; Fabregat and Caballero-Diaz, 2018) MMPs secreted by TAMs do not only release growth factors deposited in extracellular fibers but are also important for the degradation of the basement membrane and ECM, thereby enabling invasive growth and tumor cell migration. (Hynes, 2009; Yan et al., 2018) Moreover, CCL22 produced by CD163^+^ TAMs binds to CCR4 on tumor cells which induces EMT and promotes HCC invasiveness in patients. (Yeung et al., 2015) TNF-α is another major pro-metastatic factor secreted by TAMs. (Kim et al., 2009) A role for TNF-α in promotion of EMT was shown in CCA and hepatoma cell lines by increasing the expression of Snail and reducing E-Cadherin, and stabilizing ß-Catenin. (Techasen et al., 2012; Chen et al., 2019b) Interestingly, TAM-derived TNF-α was also suggested to be involved in EMT-independent mechanisms for metastasis, characterized by CXCR4-overexpressing endothelial cells evident in murine and human HCC termed “vessels that encapsulate tumor clusters” (VETC). (Xu et al., 2017; Meng et al., 2018) VETC consist of nests of primary tumor cells surrounded by a sinusoidal network. Using a xenograft and an orthotopic HCC model in mice it was shown that those clusters were able to enter the bloodstream and metastasize within the liver and to the lung independently of Snail or Slug expression. (Fang et al., 2015) In summary, TAMs do not only support tumor growth, but also facilitate tumor cell invasiveness and metastasis.

## 5 Finding new macrophage-targeting therapies for NASH-HCC

NASH and HCC and, consequently, NASH-driven HCC, represent clinical challenges due to the current lack of effective therapeutic interventions. The multi-kinase inhibitor sorafenib has been the standard of palliative care for advanced HCC for decades, although sorafenib beneficial effects were limited to about 3 months of extended survival as compared to the best supportive care, and came with a high frequency of adverse events. (Forner et al., 2012; Pang et al., 2022) One of the most described immune escape mechanisms relies on the expression of programmed-death ligand 1 (PD-L1) by tumor- and immunosuppressive immune cells. Antibodies blocking the interaction between PD-L1 and its receptor PD-1, expressed by cytotoxic CD8^+^ T cells, prevented T cell anergy and restored anti-tumor activity, and have been approved for HCC treatment. (Llovet et al., 2021) More recently, a combination therapy of the PD-L1 blocking antibody Atezolizumab and the VEGF neutralizing antibody Bevacizumab resulted in an improvement of overall and progression-free survival superior to sorafenib, and thus represent the current standard of care as first line therapy in patients with advanced HCC. (Finn et al., 2020; Llovet et al., 2021) However, a recent study consisting of a meta-analysis of three large randomized controlled phase III trials of immune checkpoint therapies in patients with advanced HCC, suggested that only patients with viral hepatitis-related HCC benefited from PD-(L) 1-targeted immunotherapy, while patients with non-viral HCC may not. A small cohort of patients with NAFLD-related HCC displayed even worse overall survival, possibly linked to the unique inflammatory immunopathology of NAFLD/NASH. (Dudek et al., 2021; Pfister et al., 2021).

**FIGURE 2:**
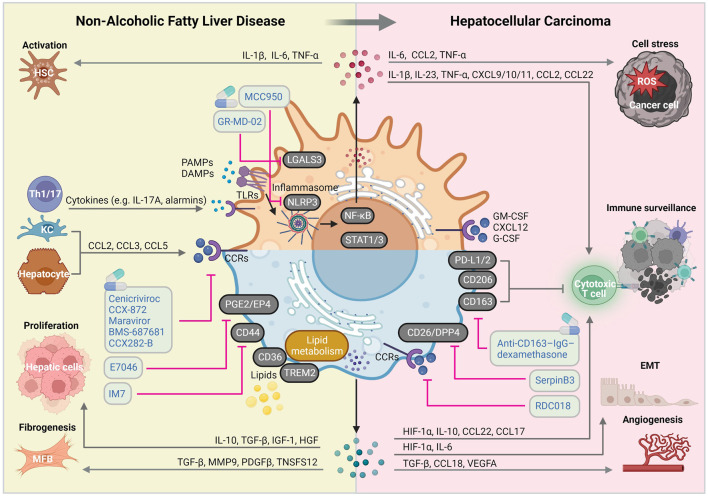
Macrophage-centered view on the molecular mechanisms implicated in NAFLD and HCC pathogenic pathways. Liver macrophages are involved in virtually all NAFLD and HCC-related pathogenic signaling pathways, giving them a central role of disease course orchestrators, with sometimes contradictory beneficial and detrimental functions. Abbreviations, CCRs, chemokine receptors; DAMPs, damage-associated molecular patterns; EMT: epithelial-mesenchymal transition; HSC, hepatic stellate cell; KC, Kupffer cell; IL, interleukin; MFB, myofibroblast; PAMPs, pathogen-associated molecular patterns; ROS, reactive oxygen species; TLRs, toll-like receptors. Created with BioRender.com.

Given that myeloid cells (especially macrophages) play predominant roles in the pathogenesis and progression of NAFLD, particular therapeutic approaches targeting macrophages rather than lymphoid cells in NAFLD-HCC might prove to have better clinical outcomes. In this review, we elaborated on the peculiar hepatic inflammatory milieu during NAFLD progression and transition to HCC. In a nutshell, the current dogma is that prolonged inflammatory and fibrogenic responses fueled by loss of KC immunotolerant functions, increases MoMF recruitment and HSC activation, drives liver disease progression and ultimately, liver cancer. (Kumar et al., 2021) Therefore, understanding key molecular alterations in macrophage-associated inflammation, fibrosis and carcinogenesis is crucial for developing new therapeutic strategies for NAFLD/NASH and NASH-HCC (Figure 2).

### 5.1 Targeting recruitment of monocyte-derived macrophages

The recruitment of MoMFs actively participates in NAFLD/NASH progression, and is largely dependent on an array of chemokine receptors (e.g., CCR2, CCR5, CXCR3). (Tomita et al., 2016; Tacke, 2017; Tacke, 2018) Thus, MoMF recruitment blockade is regarded as a promising therapeutic approach in the management of disease progression.

Intensive studies have suggested targeted chemokine interference as a therapy for NAFLD/NASH. For instance, treatment with C-C chemokine receptor (CCR) antagonists to reduce infiltration of inflammatory leukocytes have been proposed as a therapy for NASH. (Parthasarathy and Malhi, 2021; Zhang and Yang, 2021) Cenicriviroc (CVC) is an oral dual CCR2/CCR5 antagonist, which has shown promising results in murine NAFLD models and was further evaluated in NASH-related clinical trials. (Friedman et al., 2018; Tacke, 2018) The phase IIb clinical trial revealed that the one-year CVC treatment demonstrated improvement in fibrosis and no worsening of NASH compared with placebo. *In vitro* studies on TGF-β-stimulated primary mouse HSCs indicated that fibrogenic gene signatures could be directly suppressed by CVC. (Kruger et al., 2018) Furthermore, CVC ameliorated insulin resistance, hepatic inflammation, and fibrosis attributed to an efficient inhibition of CCR2^+^ monocyte recruitment. (Krenkel et al., 2018; Luci et al., 2020) CCR5 inhibition by CVC could hamper the activation, migration and proliferation of HSCs. Of note, *Ccl2* was also suggested to be expressed by fibroblasts in the steatotic mouse liver. (Guilliams et al., 2022) CVC treatment is also reported to effectively inhibit the migration of primary mouse MoMFs and lymphocytes (e.g., NK, CD4^+^ and CD8^+^ T cells). (Puengel et al., 2017; Huh et al., 2018) However, although both preclinical and phase 2 clinical studies were promising, CVC trials were interrupted in phase 3 due to a lack of efficacy in treating NASH upon a planned interim analysis (AURORA NCT03028740). (Anstee et al., 2020) Nonetheless, recent studies also provided evidence on alternative CCR2 and/or CCR5 inhibitors in NAFLD/NASH murine models that may represent more effective alternatives. The CCR2 inhibitor CCX-872 was shown to attenuate the infiltration of CD11b^+^CD11c^+^F4/80^+^ monocytes into the liver, thus improving glycemic control and liver inflammation, injury and fibrosis in a murine NAFLD model (high-fat/high-fructose diet). (Parker et al., 2018) Alternatively, the CCR5 inhibitor Maraviroc was able to arrest cell proliferation and decrease collagen production in a human HSC line. (Coppola et al., 2018) More recently, the novel dual CCR2/5 inhibitor BMS-687681 was proved to block hepatic infiltration of inflammatory monocytes in murine NASH models. (Puengel et al., 2022b) In addition to CCR2 and CCR5, the effect of chemokine blockade was investigated on several other chemokine axes. Administration of CCX282-B (a CCR9 antagonist) hampered the development of steatohepatitis, making it a promising candidate treatment for NASH patients. (Morikawa et al., 2021) Deactivation of liver CX3CL1/CX3CR1 signaling was shown to dampen NASH progression. (Ni et al., 2022)

CD44 is known as a cell-surface protein mainly expressed by immune cells. Human and experimental data suggest CD44 as a key player in NAFLD to NASH progression. CD44 promotes hepatic macrophage infiltration and polarization towards pro-inflammatory phenotypes, hence CD44-deficient macrophages were prone to polarize to anti-inflammatory phenotypes. Therefore, targeting CD44 may be taken as a potential therapeutic strategy. (Patouraux et al., 2017) In a high-fat diet (HFD) mouse model, IM7 (an anti-CD44 monoclonal antibody) injection suppressed fasting blood glucose levels, weight gain, liver steatosis, and insulin resistance, even superior to metformin and pioglitazone. (Kodama et al., 2015) These studies indicate that interfering with MoMF recruitment exerts promising roles for the prevention of NAFLD/NASH progression.

### 5.2 Targeting liver macrophage activation in NASH

Several approaches have been investigated for interfering with liver macrophage activation, such as targeting inflammatory signaling pathways like NF-kB, apoptosis signal-regulating kinase 1 (ASK1), JNK, or p38. Promising results showed improvement of steatohepatitis, liver fibrosis and HCC. (Weiskirchen and Tacke, 2016; Tacke, 2017) Selonsertib (an ASK1 inhibitor) treatment has been shown to influence hepatocyte metabolism and macrophage activation. Indeed, Selonsertib was determined to reduce liver fibrosis in NASH patients with advanced fibrosis (stage 2-3) in a randomized phase 2 trial. (Loomba et al., 2018) However, the further phase III clinical trial including more than 800 participants concluded that forty-eight weeks of Selonsertib monotherapy had no antifibrotic effects in NASH patients with bridging fibrosis or cirrhosis. (Harrison et al., 2020) Intriguingly, blockade of NLRP3 inflammasome activation in KCs and MoMFs contributed to the amelioration of NASH, by attenuating hepatic lipid accumulation. (Huang et al., 2021) MCC950 (a NLRP3 selective inhibitor) attenuated IL-1β production through inflammasome suppression, which improved NAFLD pathology and fibrosis in obese diabetic mice. (Mridha et al., 2017) STING functions as a mitochondrial DNA sensor in the KCs of liver under lipid overload and induces NF-κB-dependent inflammation in NASH. (Yu et al., 2019) The Macrophage scavenger receptor 1 (MSR1, CD204), which is overexpressed in hepatic lipid-laden foamy macrophages, plays a critical role in lipid-induced inflammation. (Govaere et al., 2022) The anti-CD163–IgG–dexamethasone strategy was applied on a rat high-fructose NASH model leading to significant reduction of inflammation, hepatocyte ballooning, fibrosis, and glycogen deposition. (Svendsen et al., 2017) E7046 (a PGE2/EP4 antagonist) significantly inhibited HSC autophagy mediated by anti-inflammatory macrophages, thus improving liver fibrosis and histopathology in NAFLD mice. (Cao et al., 2022) According to evidence from animal models, dopamine receptor D2 antagonism promotes liver regeneration over fibrosis, by selectively mediating fibrogenic crosstalk between macrophages and the vascular niche. (Qing et al., 2022) p38α-deficiency in macrophages resulted in attenuated hepatic steatosis, due to reduced secretion of pro-inflammatory cytokines (TNF-α, CXCL10 and IL-6). (Zhang et al., 2019c) Overall, although clinical benefits remain debatable, important links have been established between macrophage activation profiles and NAFLD/NASH progression from multiple preclinical studies.

### 5.3 Targeting macrophages in HCC

The liver macrophage pool is highly diverse in both pre- and malignant liver diseases. Nonetheless, several TAM-targeting therapies have been investigated. Therapeutic blocking of the CCL2/CCR2 axis counteracts the tumor-induced immunosuppression and leads to the activation of a CD8^+^ T cell anti-tumor response, attributed to the inhibition of MoMF infiltration and TAM polarization. The results suggest the CCL2/CCR2 antagonist RDC018 as a novel treatment of HCCs. (Li et al., 2017a) CD26/DPP4 was shown to aggravate immunosuppression in liver and adipose tissue *via* dysregulation of macrophage polarization. Thus, CD26/DPP4 targeting strategies, such as SerpinB3 (a DPP4 inhibitor) may serve as therapeutic approaches for NASH-associated HCC. Myeloid-specific IRE1α deletion results in functional alterations in hepatic macrophages and dampens NASH-HCC development. (Van Campenhout et al., 2020) Inhibiting APOC1 can promote the polarization of TAMs towards inflammatory macrophages *via* the ferroptosis pathway, thereby restoring an antitumor immune microenvironment and improving anti-PD1 immunotherapy for HCC. (Hao et al., 2022) Serum IgA levels were associated with fibrosis progression and HCC development. In line, *in vivo* inhibition of IgA signaling decreased the number of tumor-infiltrating IgA^+^PD-L1^high^ macrophages and increased the infiltration of CD69^+^CD8^+^ T cells, eventually leading to anti-tumoral effects in a Cell-Derived tumor Xenograft (CDX, Hepa 1-6 cells) model. (Sung et al., 2022) Apart from selective inhibitors, other families of chemicals/drugs were included in macrophage-related therapeutic HCC studies. Bufalin for instance, suppresses HCC by reversing the polarization of TAMs towards tumor-inhibitory macrophages, activating a T cell-driven anti-tumor immune response. (Yu et al., 2022) Similarly, metformin significantly drive beneficial macrophage polarization and T cell infiltration, which suggests therapeutic effects of metformin on tumor surveillance. (de Oliveira et al., 2019)

## 6 Concluding remarks

In recent years, in-depth characterization of physiological and pathological mechanisms at the singular cell level led to unprecedented insights into cellular diversity in a complex microenvironment. Consequently, earlier macrophage classification (e.g., the dichotomous M1/M2 paradigm) appear outdated. Indeed, the granularity of recent datasets allowed us to recognize a wide range of diversity, with complementary or opposite roles observed simultaneously in multiple subpopulations. Recent data also emphasized the importance of spatial contextualization, providing crucial hints into the effective functions of defined myeloid subsets. Nevertheless and despite such diversity, numerous studies also highlighted the central roles of liver macrophages as key orchestrators of not only the immune response, but also of disease progression, from initiation to malignancies. Thus, targeted strategies aiming at shaping a specific macrophage landscape, or spatially-resolved interventions aiming at favoring beneficial macrophage populations must be further explored. There is much left to discover on the events marking the transition between a *hot*, largely pro-inflammatory immune landscape observed during liver disease progression, and a *cold* tumor immune microenvironment. Thus, therapies should aim at limiting the effects of an over-reacting immune system on one hand, while preventing tumor favorable immunological conditions.

## Abbreviations

ARG1, Arginase 1; CCA, Cholangiocarcinoma; CD, Cluster of differentiation; ECM, Extracellular matrix; EMT, Epithelial-mesenchymal transition; FAO, Fatty acid oxidation; HCC, Hepatocellular carcinoma; HFD, High fat diet; HGF, Hepatocyte growth factor; HIF-1α, hypoxia-inducible factor-1 alpha; HSC, Hepatic stellate cell; IL, Interleukin; KC, Kupffer cell; MDSC, Myeloid-derived suppressor cell; MerTK, Mer Tyrosine Kinase; MMP, Matrix metalloproteinase; MoMF, Monocyte-derived macrophage; NAFLD, Non-alcoholic fatty liver disease; NASH, Non-alcoholic steatohepatitis; ROS, Reactive oxygen species; SIRPα, Signal-regulatory protein alpha; TAM, Tumor-associated macrophage; TGF-β, Transforming growth factor-beta; TME, Tumor microenvironment; TNF-α, Tumor necrosis factor alpha; TREM, Triggering receptor expressed on myeloid cells.
